# A Brief History of Cardiomyoplasty: Worth Another Look?

**DOI:** 10.31083/j.rcm2305159

**Published:** 2022-04-28

**Authors:** John A. Elefteriades

**Affiliations:** ^1^Aortic Institute at Yale-New Haven Hospital, Yale University School of Medicine, New Haven, CT 06510, USA

**Keywords:** cardiomyoplasty, heart failure, cardiac assist, skeletal muscle, muscle conditioning, accessory ventricle

## Abstract

This article reviews the concept and extensive experimentation done over two decades ago to convert and apply skeletal muscle for cardiac assistance—so called “cardiomyoplasty”. Skeletal muscle was either wrapped around a failing heart or fashioned into accessory cardiac pumping chambers. Although the era of cardiomyoplasty came to an end when the cardiac wraps were found ineffective, the concept of independent accessory skeletal muscle ventricles may be worth another look.

## 1. Introduction

Cardiac specialists should be made aware of the vigorous research done in the field of “cardiomyoplasty” in the late 1980s and early 1990s. The current generation of trainees is not even aware of this once burgeoning field—one which may well be worthy of a further look as a treatment for advanced heart failure.

## 2. Cardiac vs. Skeletal Muscle

There is only one muscle in the body capable of the tremendous task of beating continuously minute-by-minute, hour-by-hour, day-by-day for a lifetime: the heart. Thanks to the electrical syncytium, each and every ventricular muscle cell contracts with each and every heartbeat. When considered in this way, the task of perfusing the body continuously is truly remarkable. Our biceps or our triceps cannot even come close. No skeletal muscle can do so. The cardiac muscle has become specialized to permit continuous performance. This is achieved by a complete transformation of enzyme systems from those of skeletal muscle.

One might argue that the diaphragm comes close to cardiac muscle in its endurance, and certainly it does. However, not every radicle of the phrenic nerve fires, and not every diaphragm muscle fiber contracts, with each breath. Rather, the brain rotates the duty cycle intricately among different groups of muscle fibers with each breath—so that some fibers are resting and replenishing while others work.

## 3. Diaphragm Pacing—Converting Diaphragm Muscle Cells to A Tireless State, Like Cardiac Muscle

The author’s mentor, Dr. William W.L. Glenn, was the pioneer of diaphragm pacing—for central hypoventilation or high quadriplegia. In those conditions, the lower motor neurons of the phrenic nerve are intact, but the upper motor neurons are dysfunctional and do not tell the phrenic to stimulate the diaphragm. Dr. Glenn learned in the 1960s that the diaphragm could indeed be paced by electrical stimulation of the phrenic nerve. However, because pacing depolarized every fiber in the phrenic nerve with each breath, all diaphragmatic muscle cells were stimulated simultaneously and continuously over time. This was intolerable, as the diaphragm tired after just a few minutes of such stimulation. However, the diaphragm could be trained by a program of gradually progressive stimulation. Over months of gradual training, the diaphragm became tireless and capable of tolerating pacing 24-hours a day, seven days a week. In essence, Dr. Glenn, via gradual stimulation, was transforming diaphragmatic muscle to a cardiac muscle equivalent. This transition involved conversion from fast-twitch easily fatiguing muscle cells to slow-twitch fatigue-resistant muscle cells.

## 4. Skeletal Muscle Transformation for Cardiopyoplasty

As Dr. Glenn’s disciple during my residency, I accumulated robust clinical experience transforming diaphragmatic muscle in this way. In that era, our cardiac surgical team did not only all the diaphragm pacemakers, but also all the implantable cardiac pacemakers, including both epicardial and transvenous. So, Dr. Glenn’s training gave me advanced knowledge of pacing techniques. Armed with experience training diaphragm muscle and pacing the heart, I was naturally drawn in to the field of cardiomyoplasty.

The process of stimulating to achieve transformation of diaphragm or skeletal muscle to a tireless state required not just a single electrical stimulus, but rather a train of stimuli delivered in quick succession. Pacemakers were developed that mirrored our diaphragm-specific train of stimuli, now for the cardiomyoplasty application. Under conditioning with pulse train stimulation, skeletal muscle underwent (via enzymatic, morphologic, and functional alterations) a progressive, elegant, and reliable transformation toward a “tireless” phenotype.

## 5. Initial Experiments

Throughout the world, especially in the United States and Europe, teams went to work training cardiac muscle. Great pioneers from the muscle physiology world and the cardiac surgical world cooperated in these experiments. Key experts working in this field included Chiu, Chachques, Carpentier, Acker, Stephensen, and many others. The principles of optimal training of skeletal muscle to resemble cardiac muscle were quickly elucidated.

**Fig. 1.**
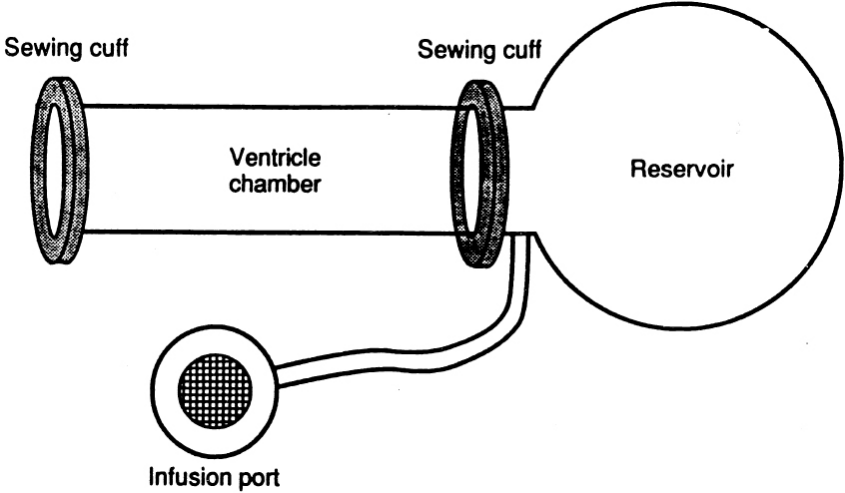
**Silicone elastomer mandrel used for creation of skeletal muscle ventricles**. Note the cylindrical portion, the spherical portion, and the Dacron sewing cuffs (for attachment to skeletal muscle wraps). The mobilized latissimus muscle was wrapped in layers around the cylindrical portion. Reprinted with permission from Reference [1].

**Fig. 2.**
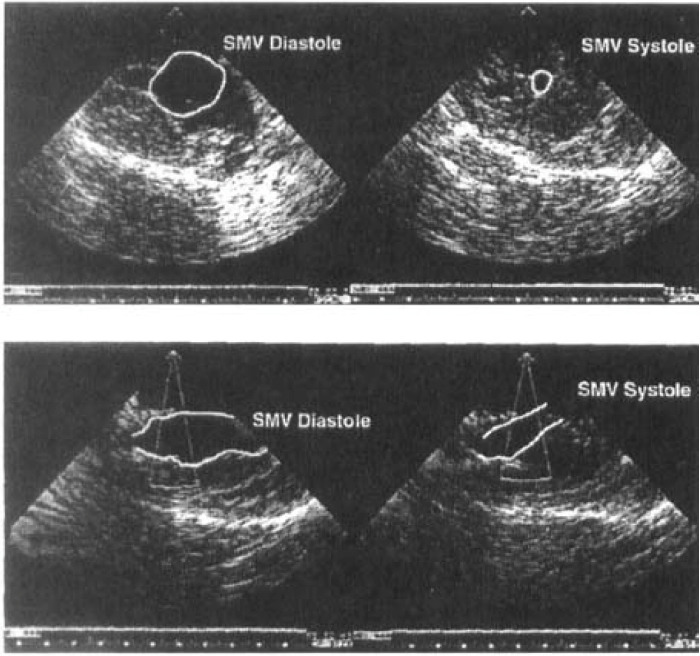
**Echocardiographic examination of the skeletal muscle ventricle (SMV) functioning in continuity with the circulation**. Top shows short-axis view, and bottom shows long-axis view. Note vigorous contraction between systole and diastole. Reprinted with permission from Reference [3].

**Fig. 3.**
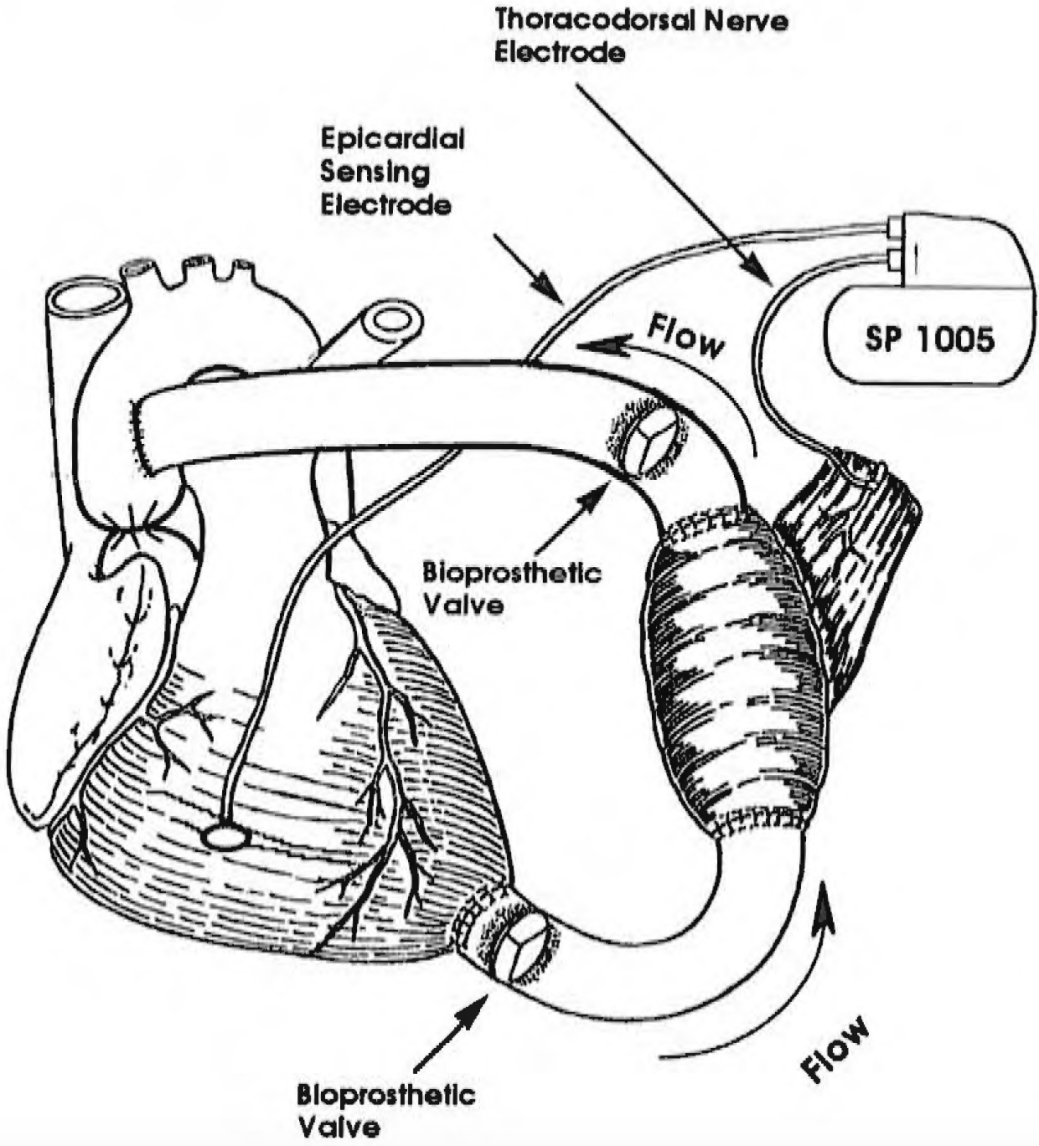
**Skeletal muscle ventricle (SMV) connected like an LVAD (left ventricular assist device) between the left ventricular apex and the ascending aorta, to assist the left ventricle**. Note inlet and outlet valves for the SMV. Reprinted with permission from Reference [3].

In our laboratory, experimentation proceeded with the collaboration and guidance of experts from multiple disciplines. David Franciscelli, a Bakken award winning engineer from Medtronic, provided pacing and engineering expertise. Preeminent plastic surgeon Dr. Stephen Ariyan supervised harvesting of the lattisimus dorso muscle and its fashioning into a neo-ventricle. Cardiac surgeon Dr. George Letsou, as well as many talented residents, participated as we constructed cylindrically-shaped skeletal muscle ventricles in our laboratory. We wrapped layers of latissimus dorsi muscle around a plastic mandrel [1, 2, 3, 4] (See Fig. 1, Ref. [1], Fig. 2, Ref. [3], Fig. 3, Ref. [3]). Of course, we left the latissiumus dorsi muscle pedicled on its blood and nerve supply (thoracodorsal artery and nerve). We “matured” these neo-ventricles while administering progressive pacing over weeks. By that point, the ventricles were no longer just wraps like “roll-ups”, but rather muscle layers adherent and shaped into a discrete, self-maintaining neo-ventricle.

**Fig. 4.**
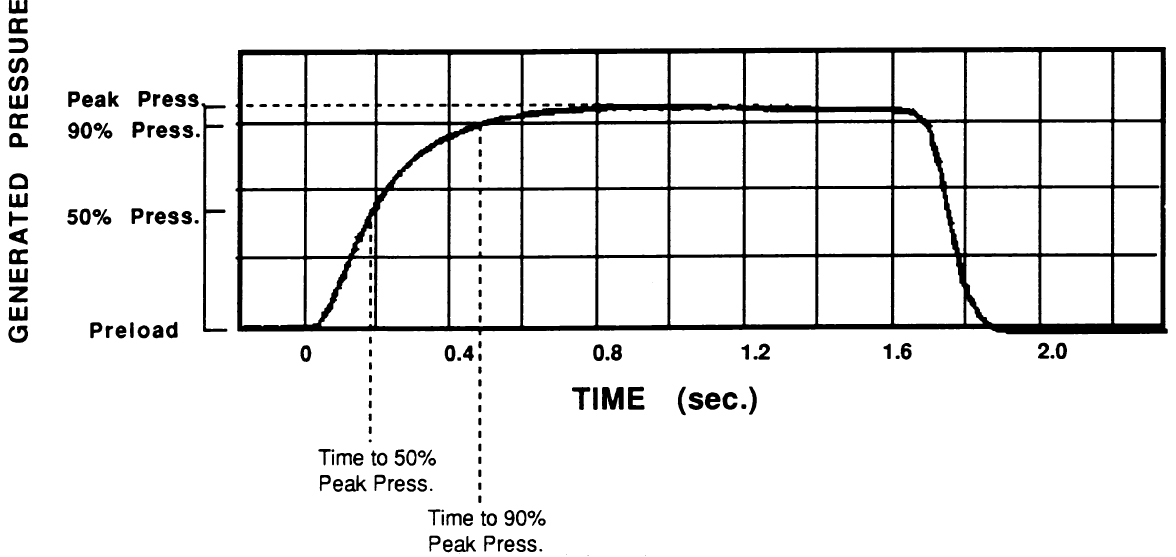
**Note sustained pressure generation by skeletal muscle ventricle**. Reproduced with permission from Reference [1].

**Fig. 5.**
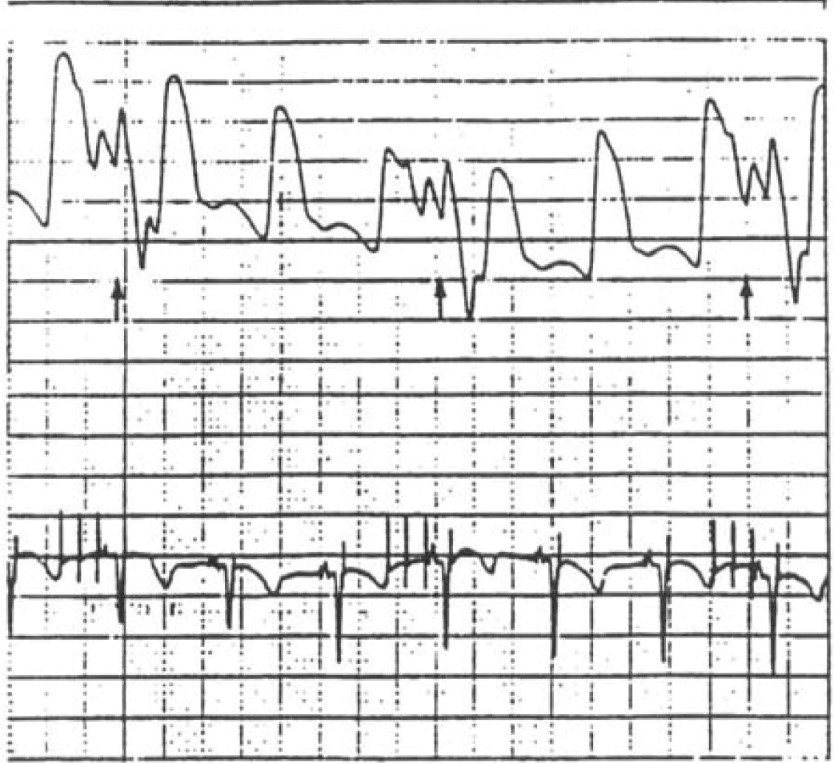
**SMV connected in counterpulsation mode. Note diastolic pressure generation, just like a mechanical intra-aortic balloon pump (IABP)**. Note also the pulse train electrical stimuli (series of three vertical spikes in EKG trace). SMV “IABP” is in 1:3 timing mode.

After weeks of maturation, we connected the neo-ventricles in parallel with the native aorta, usually with inlet and outlet mechanical valves. These conditioned skeletal muscle ventricles were capable of generating pressure quite effectively and reliably, and in a sustained fashion. See Fig. 4, Ref. [1]. We met with moderate success in these efforts. We were quite encouraged that these experiments could lead to accessory ventricles made entirely of native muscle which could benefit patients with advanced heart failure. See Fig. 5, Ref. [1].

Many centers worldwide pursued similar programs of investigation, advancing similarly or even beyond our experiments at Yale.

## 6. Going for a Grand Slam rather than a Base Hit

As these skeletal muscle ventricles (SMV) experiments were proceeding, other groups were simply wrapping a layer of latissimus dorsi muscle around the heart, rather than constructing the more complex independent accessory skeletal muscle chambers. The extra “squeeze” of the trained skeletal muscle would, it was postulated, augment the native cardiac contraction. As this model of application of skeletal muscle did not involve creation of any chambers or any major vascular anastomoses (or inflow and outflow valves), this was a much simpler paradigm. Excitement was literally rampant throughout the muscle physiology, cardiology, and cardiac surgical communities.

At that time, the National Institutes of Health, reflecting the worldwide excitement, decided to fund a clinical study of wrapping conditioned muscle around the human heart.

First-in-man experiments with the wrap cardiomyoplasty were performed on over 1500 patients. Initial reports were encouraging, with multiple early signs of clinical amelioration reported [5, 6, 7]. After several years, however, it became abundantly clear (via the C-SMART Trial: Cardiomyoplasty-Skeletal Muscle Assist Randomized Trial) that patients were not benefitting in any reliable or significant fashion, neither in terms of hemodynamics nor in terms of survival. Wrapping of the heart with skeletal muscle ceased worldwide [6, 7].

Much to my dismay, governmental and commercial funding of cardiomyoplasty experiments suddenly dried up—completely and permanently. Medtronic stopped supporting the previously promising laboratory investigations, even those exploring independent SMV chambers rather than simple wrapping of the heart with skeletal muscle. The era of exploration of skeletal muscle for cardiac assist came to an end.

## 7. Worth Another Look? (Personal Perspectives, J. Elefteriades, MD)

My own feeling at that time was one of disappointment. I had never been optimistic about just wrapping the heart. After all, as we in the heart failure and transplantation community know very well, the hearts become enormous at the latter stages of the heart failure continuum. I had never been optimistic about simply wrapping the heart with trained skeletal muscle. I thought that the burden of dealing with the high wall tension of those massively enlarged hearts would simply be too much.

However, I believed then, and I still believe now, that we in the cardiomyoplasty community were very nearly ready for clinical trials of accessory ventricles of trained skeletal muscle, working in parallel with the native circulation. These independent, accessory skeletal muscle ventricles were not burdened by squeezing a massively enlarged heart.

I was disappointed not to be able to continue those experiments. The whole field of conditioning skeletal muscle for cardiac augmentation ceased to exist immediately after the disappointing results of the “wrap” operation were disseminated.

I believed then, and I still believe now, that the cardiomyoplasty concept, via accessory ventricles, deserves additional investigation and continues to hold promise. Historically, I believe it was the abrupt move to wrapping the heart, a potential quick solution, rather than continuing to work on the more promising creation of accessory ventricles, which doomed the original clinical trials.

What might be a contemporary path forward for cardiomyoplasty? I would envision starting a renewed exploration of cardiomyoplasty, benefitting from the years of prior concerted experimentation. I would favor taking the accessory ventricle approach, not a wrapping approach. Additional experimental work would be required before clinical application in humans. A two-stage surgical approach would likely be beneficial: Stage 1, “Construction, Moulding, and Training”, would involve harvesting the latissimus dorsi muscle, training it gradually over weeks with pulse-train stimulation, and allowing the unloaded accessory ventricle to take shape over a compressible mandrel. Stage 2, “Connection to the circulation” would then be done, in any of a variety of configurations–perhaps optimally in a left ventricle-to-aorta configuration. Ultimately, a human trial would be required. Cardiomyoplasty could be added on top of current state-of-the-art conventional medical therapy, which has advanced dramatically in the decades since cardiomyoplasty was originally explored. Comparison of outcomes between surgical cardiomyoplasty and optimal medical therapy could provide clear, unambiguous evaluation of any benefit from cardiomyoplasty, and its magnitude. Detailed echocardiographic imaging, as well as MRI (magnetic resonance imaging), could provide objective evidence of benefit, above and beyond any clinical improvement.

Despite devoting many of the intervening years to treating patients with mechanical support devices and transplantation, I still believe that further research in constructing skeletal muscle ventricles may ultimately lead to a more “natural” augmentation “device”, made entirely of native cells—without the issues of blood contact with foreign materials or the danger of rejection of allograft hearts.
